# The Influence of the Capping Step During Solid-Phase Phosphoramidite Synthesis of Oligonucleotides on Synthetic Errors in Oligonucleotides

**DOI:** 10.3390/molecules31010094

**Published:** 2025-12-25

**Authors:** Kristina I. Yakovleva, Ivan M. Pereverzev, Andrey A. Kechin, Ulyana A. Boyarskikh, Maxim L. Filipenko, Georgiy Y. Shevelev, Yuliya V. Sherstyuk, Ilya S. Dovydenko

**Affiliations:** Institute of Chemical Biology and Fundamental Medicine SB RAS, Novosibirsk 630090, Russia

**Keywords:** phosphoramidite oligonucleotide synthesis, G to A substitutions, guanine modification, capping

## Abstract

Errors in de novo synthesized DNA can originate from the oligonucleotides used during assembly. Oligonucleotides may contain substitutions, deletions, and insertions resulting from either incomplete reactions at individual steps of the phosphoramidite synthetic cycle or various side reactions. In this study, we quantitatively assessed errors in both gene constructs assembled from synthetic oligonucleotides by Sanger sequencing and in synthetic oligonucleotides by NGS. Our data demonstrate that side reactions involving carboxylic acid anhydrides during the capping step of oligonucleotide synthesis lead to the modification of guanine residues. This guanine modification subsequently results in the accumulation of G to A substitutions in the final gene constructs. We show that the error rate can be reduced by replacing the standard acetic anhydride-based capping mixture with anhydrides of carboxylic acids weaker than acetic acid. Furthermore, a more significant reduction in errors is achievable by using capping reagents based on phosphoramidite chemistry.

## 1. Introduction

Synthetic oligonucleotides are widely used across diverse fields such as molecular biology, genetic engineering, biotechnology, and medicine—both for diagnostics and therapeutics [1,2,3,4]. Oligonucleotides are also in high demand for synthetic biology applications, particularly for de novo gene assembly [5,6]. While researchers were limited to synthesizing short gene constructs only a few decades ago [7,8], the assembly of entire genomes is now feasible due to advancements in high-throughput oligonucleotide synthesis and enzymatic methods for large DNA construction [9,10]. Indeed, de novo synthesis of relatively short gene constructs spanning several kilobases (kb) is now considered a routine task. However, this routine process is complicated by the necessity to correct errors in the assembled gene sequences, which entails additional time and resources expenditures [11]. Errors in synthetic genes can originate during enzymatic gene assembly process or be inherited from the chemically synthesized oligonucleotides used [12]. Oligonucleotides may contain base substitutions, deletions, and insertions resulting from incomplete reactions during the phosphoramidite synthesis cycle or side reactions [13]. Therefore, developing strategies to reduce error rates in synthetic oligonucleotides remains a critical challenge—not only for synthetic biology and genetic engineering but also for all the aforementioned fields.

In this study, we quantitatively assessed error rates in gene constructs assembled from synthetic oligonucleotides using Sanger sequencing. Additionally, we evaluated errors in oligonucleotides synthesized under various conditions using next-generation sequencing (NGS). We also propose a modification to the standard phosphoramidite oligonucleotide synthesis cycle that reduces the frequency of specific error types.

## 2. Results and Discussion

**Scheme 1:**
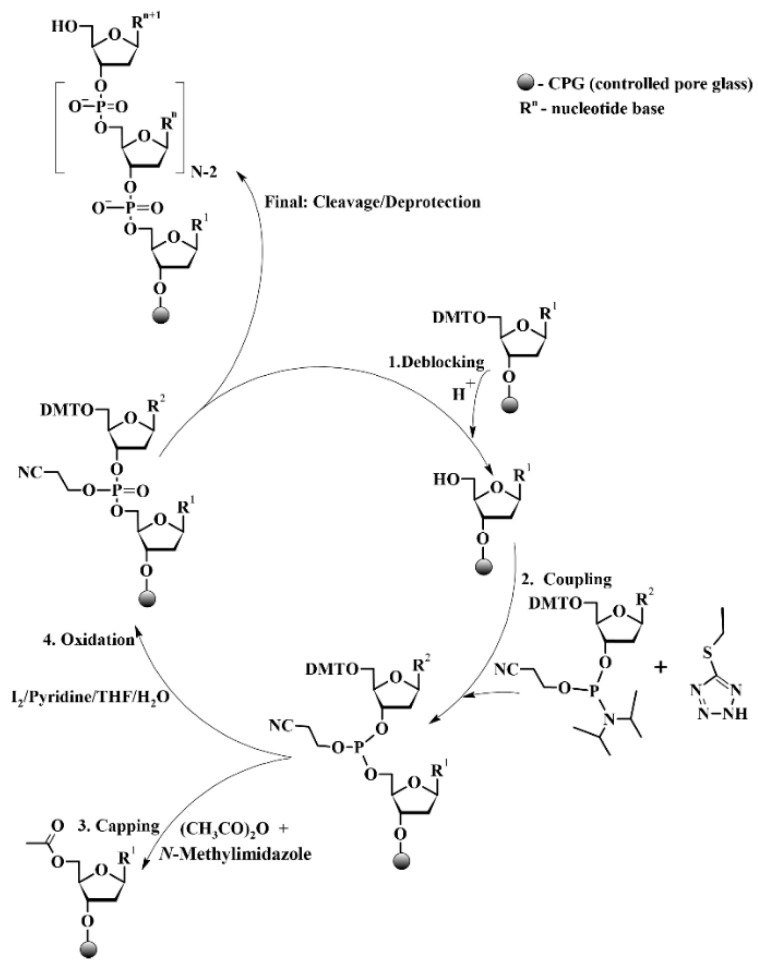
Synthetic cycle of the solid-phase oligonucleotide synthesis by phosphoramidite method. The cycle is repeated N times until the target sequence is reached.

In our laboratory, oligonucleotides were synthesized using the solid-phase phosphoramidite method (see Scheme 1), developed by M. H. Caruthers [14].

We carried out the synthesis on a 96-well DNA synthesizer ASM-2000 (Biosset, Russia) according to the following protocol, see Table 1.

Dry reagents and anhydrous solvents were used in the synthesis. For clarity, propionic anhydride (Pr_2_O) was employed in capping reagent A (CapA) in our laboratory instead of the commonly used acetic anhydride (Ac_2_O), as the latter is subject to regulatory controls in the Russian Federation. In our experience, Pr_2_O in CapA is as effective as Ac_2_O for this application. After the synthesis, CPG-bound protected oligonucleotides were treated with AMA reagent (see Table 1). The resulting deprotected oligonucleotides were then precipitated in ethanol with 0.3 M NaCl. The dried precipitates were dissolved in water and used directly, without further purification, for the assembly of long DNA sequences via polymerase cycling assembly (PCA) [15].

**Figure 1:**
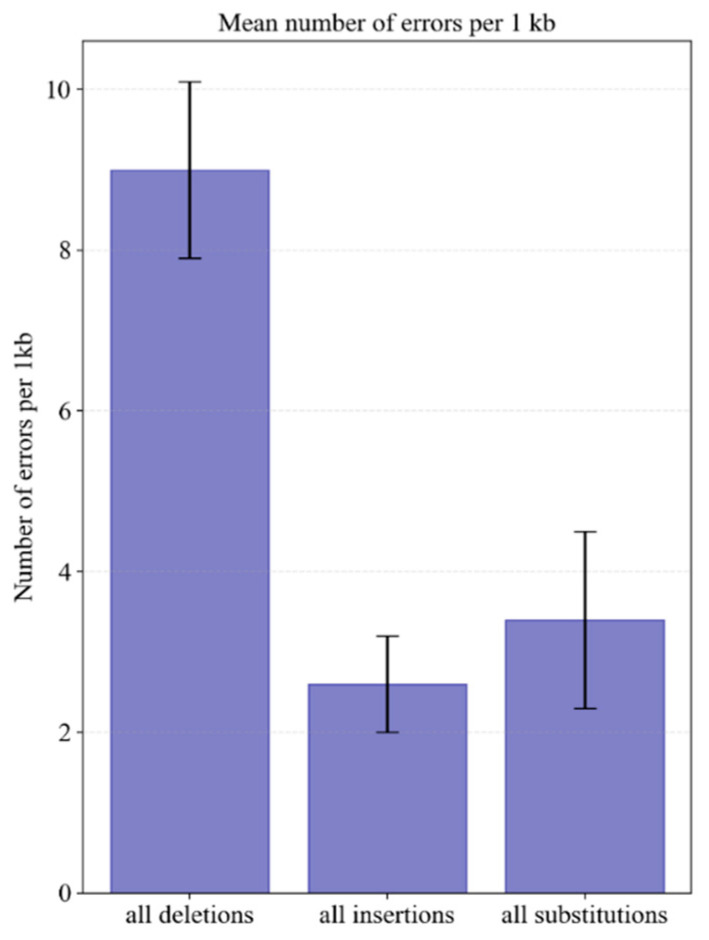
Mean number of errors per 1 kb for assembled T5 exonuclease gene. Errors are grouped into three types: deletions, insertions, and substitutes.

Synthesis accuracy was assessed via Sanger sequencing, using the T5 exonuclease gene as a model (see Supplementary File S1.Fasta S1). The gene sequence was divided into partially overlapping oligonucleotides using GeneCut software (http://genecut.unipro.ru/) (access date: 25 November 2024) (see Supplementary File S1.Table S1) [16]. These oligonucleotides were synthesized as described above and used for PCA. The assembled T5 exonuclease gene was amplified and cloned into the *pUC19* vector. Recombinant vectors were transformed into *E. coli* (NovaBlue) cells, which were then plated on antibiotic-containing agar. Ten colonies containing inserts of the expected length were selected via colony PCR screening and subjected to Sanger sequencing. To account for potential variability during assembly, cloning, or host–cell variability, the entire process was independently repeated three times. A Kruskal–Wallis test [17] comparing error distributions across the three experiments revealed no statistically significant differences (*p*-value = 0.93). Detailed error types and statistical analyses are provided in Supplementary File S2. Analysis of Sanger sequencing data revealed that deletions were the most frequent error (9 ± 1.1 per kb), followed by substitutions (3.4 ± 1.1 per kb) and insertions (2.4 ± 0.6 per kb) (Figure 1, Supplementary File S2).

The high frequency of deletions, along with the presence of insertions, can be attributed to the use of crude oligonucleotide mixtures containing N-1 truncated failure sequences. Nevertheless, despite this error rate, sequencing only nine clones is statistically sufficient to obtain a correct 1 kb fragment with 90% probability (see Supplementary File S2).

**Figure 2:**
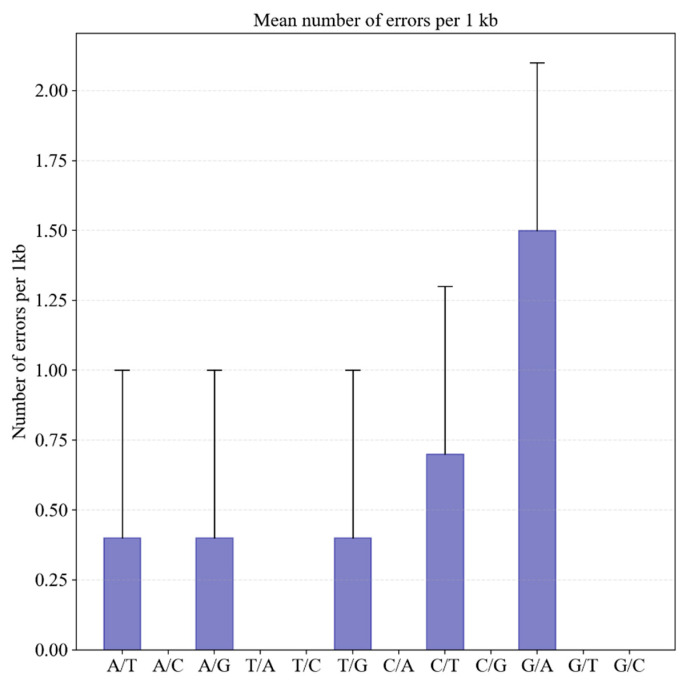
Mean number of substitution errors per 1 kb for assembled T5 exonuclease gene.

After deletions, base substitutions were the most frequent errors (Figure 1), with G/A and C/T being the predominant types (see Figure 2).

The prevalence of G/A and C/T substitutions may result from: (i) mechanical defects in the DNA synthesizer leading to incorrect phosphoramidite dispensing; (ii) polymerase-induced errors during PCA or gene amplification; or (iii) side reactions involving nucleobases during oligonucleotide synthesis or post-synthetic processing. We excluded accidental cross-contamination with dA or dT phosphoramidites as such an event would produce comparable levels of T/A, C/A, A/T, and G/T substitutions, which were not observed. Indeed, in the assembly of the T5 exonuclease gene, we did not detect any T/A, C/A, or G/T substitutions at all (Figure 2, Supplementary File S2). We also ruled out polymerase-related errors during PCA and amplification as the source of G/A and C/T substitutions. In our experiments, we used high-fidelity Phusion polymerase [18].

Therefore, we hypothesize that G/A and C/T substitutions arise from the chemical modification of cytosine and/or guanine bases during oligonucleotide synthesis or post-synthetic processing. These modified bases are misread by the polymerase during PCA, leading to the observed G/A and C/T substitutions in the final gene construct.

A similar predominance of G/A and C/T substitutions over other substitution types was observed in the assembly of the interleukin-2 (IL-2) gene and the gene encoding the Sso7d protein (see Supplementary File S2). This consistency raises the question of whether such a distribution is general or specifically associated with our synthesis protocol (Table 1).

There is a notable lack of studies providing comprehensive statistical data on the accumulation of specific error types in synthetic DNA. Such data are only occasionally reported in studies focused on error correction in assembled gene constructs. For instance, several reports indicate that, prior to correction, the majority of the mismatches in the original constructs were G:T/A:C mismatches [19,20,21]. However, this information remains difficult to interpret without precise knowledge of the specific synthesis protocols used for the constituent oligonucleotides.

In 2021, S. Filges et al. published a study evaluating errors in chemically synthesized oligonucleotides using a digital sequencing approach with unique molecular identifiers [13]. By analyzing oligonucleotides from various suppliers, the authors found that deletions were the predominant error type across nearly all batches. Substitutions were also reported, although a detailed breakdown of substitution types was not provided. The average substitution frequency across all oligonucleotide types was 0.025%, which was seven times lower than the observed deletion rate.

Later, in 2022, Y. Masaki et al. reported a quantitative assessment of errors in synthetic oligonucleotides using NGS [22]. They demonstrated that G/A substitutions accumulate depending on the capping conditions. The authors hypothesized that these substitutions result from the modification of the *O*^6^-position of guanine residues by components of the capping mixture, leading to the formation of 2,6-diaminopurine (DAP). This hypothesis was indirectly supported by experiments in which standard deoxyguanosine phosphoramidites were replaced with analogs bearing modified bases, such as 7-deazaguanine and 8-aza-7-deazaguanine. The use of these modified phosphoramidites reduced the G/A substitution frequency by 10-fold for 7-deazaguanine and by 50-fold for 8-aza-7-deazaguanine, compared to standard guanine.

Several research groups have previously reported side reactions involving the guanine base during solid-phase phosphoramidite synthesis [23,24]. Specifically, Rodriguez et al. investigated the formation of side products under excessive capping conditions (86 equivalents of Ac_2_O over 15 min for cycle 1, and 17.2 equivalents over 1.5 min for cycles 2 through the penultimate cycle) [23]. Oligonucleotides synthesized under these conditions contained 9.2% of an impurity with an additional mass of +41 amu, identified via reverse-phase high-performance liquid chromatography–electrospray ionization mass spectrometry (RP-HPLC-ESI-MS). This +41 amu impurity was absent when the capping step was omitted. Based on these findings, the authors concluded that the additional mass resulted from the formation of an *N*^2^-acetyl-2,6-diaminopurine derivative induced by the excessive capping (prolonged reaction time and high reagent equivalents).

Earlier, Eadie and Davidson reported the presence of a fluorescent impurity in deprotected oligonucleotides synthesized using acetic anhydride, 4-dimethylaminopyridine (DMAP), and lutidine as capping reagents [24]. They demonstrated that this modification occurs at guanine residues, generating an impurity with an absorption spectrum in the 330–400 nm range. This fluorescent intermediate was shown to partially convert in ammonium hydroxide solution to a 2,6-diaminopurine deoxyribonucleoside. Notably, the authors also observed the accumulation of this impurity under conditions where only DMAP and lutidine in tetrahydrofuran—without acetic anhydride—were used as the capping mixture. This finding, combined with prior reports that phosphoramidite derivatives of protected nucleosides can react with the *N*^2^-protected guanine base as well as the 5′-hydroxyl group [25,26], led the authors [24] to propose that DAP nucleoside formation may originate from a phosphite triester adduct at the *O*^6^-position of guanine formed during the coupling step. Furthermore, they reported that replacing DMAP with *N*-methylimidazole (NMI) during capping completely eliminated the fluorescent product, although the amount of DAP nucleoside was reduced only 15-fold [24].

As indicated in the literature review above, the presumed cause of the G/A substitutions is modification of the *N*^2^-protected guanine residue. However, for over 35 years, a comprehensive understanding of the mechanisms underlying these modifications during solid-phase synthesis has remained elusive, beyond their general association with the capping step.

**Figure 3:**
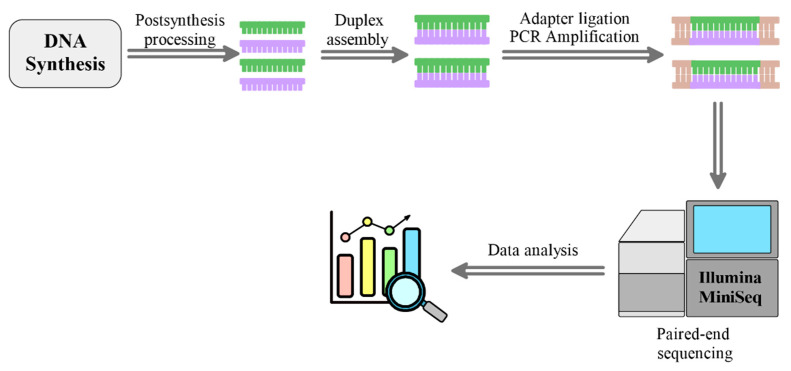
The experimental workflow consisted of four stages: synthesis of complementary oligonucleotides; post-synthetic processing, including cleavage from the solid support, deprotection, and purification via polyacrylamide gel electrophoresis (PAGE); NGS library preparation, involving duplex formation, adapter ligation, and amplification of the final constructs; and NGS followed by data analysis.

To investigate how different capping conditions influence the probability of G/A substitutions, we synthesized six pairs of 107 bp DNA duplexes and analyzed them using NGS (Figure 3).

Duplex-forming oligonucleotides were synthesized using our baseline protocol in which capping was performed with a Pr_2_O/NMI mixture (see Table 1), as well as modified protocols featuring variations in the capping step (Table 2).

**Figure 4:**
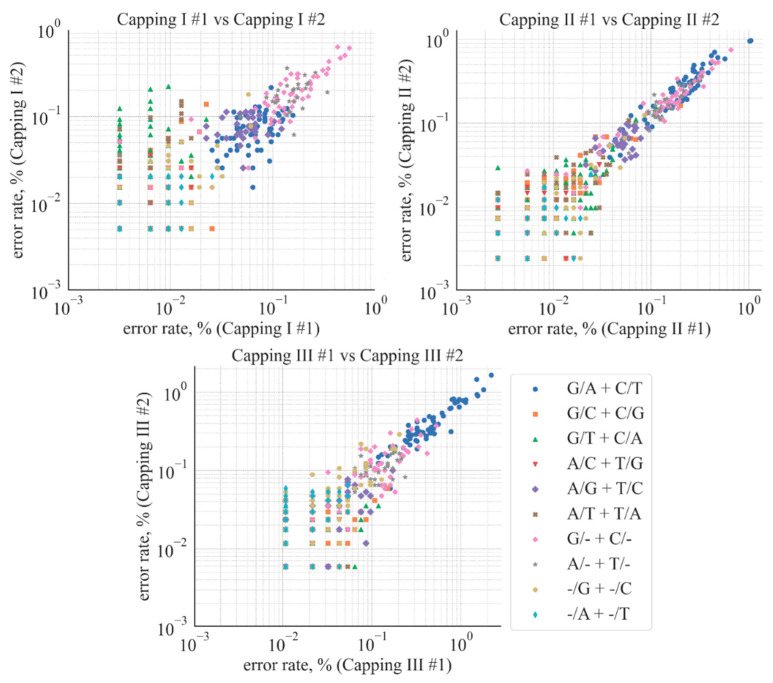
Reproducibility of observed error rates. Two (#1 and #2) 107 bp duplexes were compared. The duplexes consisted of oligonucleotides, which were synthesized independently using the same synthetic conditions.

The synthesized oligonucleotides were purified by PAGE to minimize the impact of deletions and insertions. For each capping condition, duplicate syntheses were performed for each strand of the duplex (designated as #1 and #2). The NGS results are presented in Figure 4, with the corresponding raw data provided in Supplementary File S3. A comparison between the two independent syntheses for each capping condition demonstrates the high reproducibility of the errors profile for each protocol (Figure 4).

**Figure 5:**
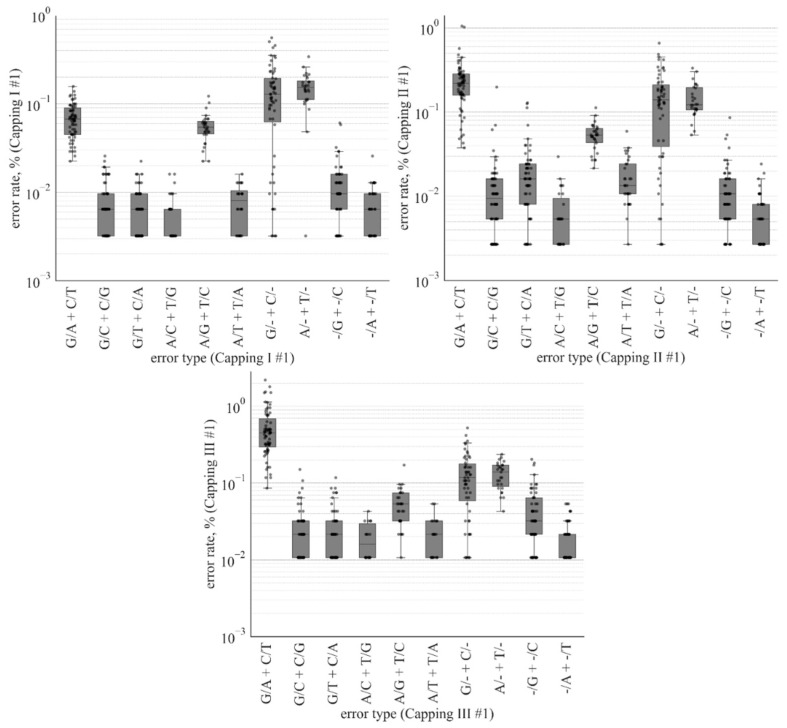
Error rates for substitutions, deletions and insertions for 107 bp duplexes. Oligonucleotide for duplex preparation were synthesized with different capping conditions: Capping I, Capping II and Capping III.

In the protocol without capping (Capping I, Table 3), deletions were the predominant error types (Figure 5). The median error probabilities for the G/− + C/− and A/− + T/− pairs were 0.128% and 0.153%, respectively.

In addition to deletions, the most frequent errors were base substitutions, specifically G/A + C/T and A/G + T/C. The median error probability for G/A + C/T substitutions was 0.067%, while for A/G + T/C substitutions, it was 0.055%.

Introduction of the capping step into the synthesis protocol significantly increases the frequency of G/A + C/T substitutions (blue circles, Figure 4 and Figure 5). For instance, using Pr_2_O/NMI (Capping II, Table 2), the G/A + C/T substitution probability rises to 0.22%, and with Pr_2_O/DMAP (Capping III, Table 2), it increases further to 0.451%. Notably, under capping conditions, G/A + C/T substitutions became more frequent than deletions, a reversal of the trend observed in Capping I (Table 2). Analysis revealed that deletions and A/G + T/C substitutions, identified as the most prevalent errors in the no-capping protocol, remained unaffected by the presence of the capping step. Based on these data, we concluded that the capping step promotes side reactions leading to guanine base modification, consistent with previously reported finding [22,23,24].

Can the frequency of G/A + C/T substitutions be modulated by altering the composition of the capping reagents? Furthermore, how do modifications to the synthesis protocol affect the magnitude of deletion errors? To address these questions, we performed a series of experiments (Figure 3) with specific adjustments. Since a key objective was to investigate the impact of capping conditions on deletion frequency, we utilized crude (unpurified) oligonucleotides for this analysis.

As chain length increases, the efficiency of all steps in the oligonucleotide synthesis cycle typically decreases, promoting the accumulation of deletions [27]. To eliminate the influence of chain length on deletion error frequency, we employed a shortened 50 bp duplex instead of the previously used 107 bp duplex. Each strand of the duplex was synthesized using both the baseline protocol (see Table 1) as well as modified protocol where the capping reagent composition, the number of capping cycles, and their order were varied (see Table 3).

For the study, acetic and propionic anhydrides in combination with *N*-methylimidazole, as well as the commercially available UniCap Phosphoramidite (Glen Research) used with 5-ethylthio-1H-tetrazole, were selected as capping reagents. The rationale for employing a phosphoramidite-derived capping agent originated from our sequencing data (Table 2, Figure 5), which show that 107-mer oligonucleotides synthesized without a capping step exhibited the lowest levels of G/A + C/T substitutions (Figure 5). Based on this observation, we hypothesized that utilizing phosphoramidite-based chemistry for capping would maintain these low substitutions frequencies.

**Figure 6:**
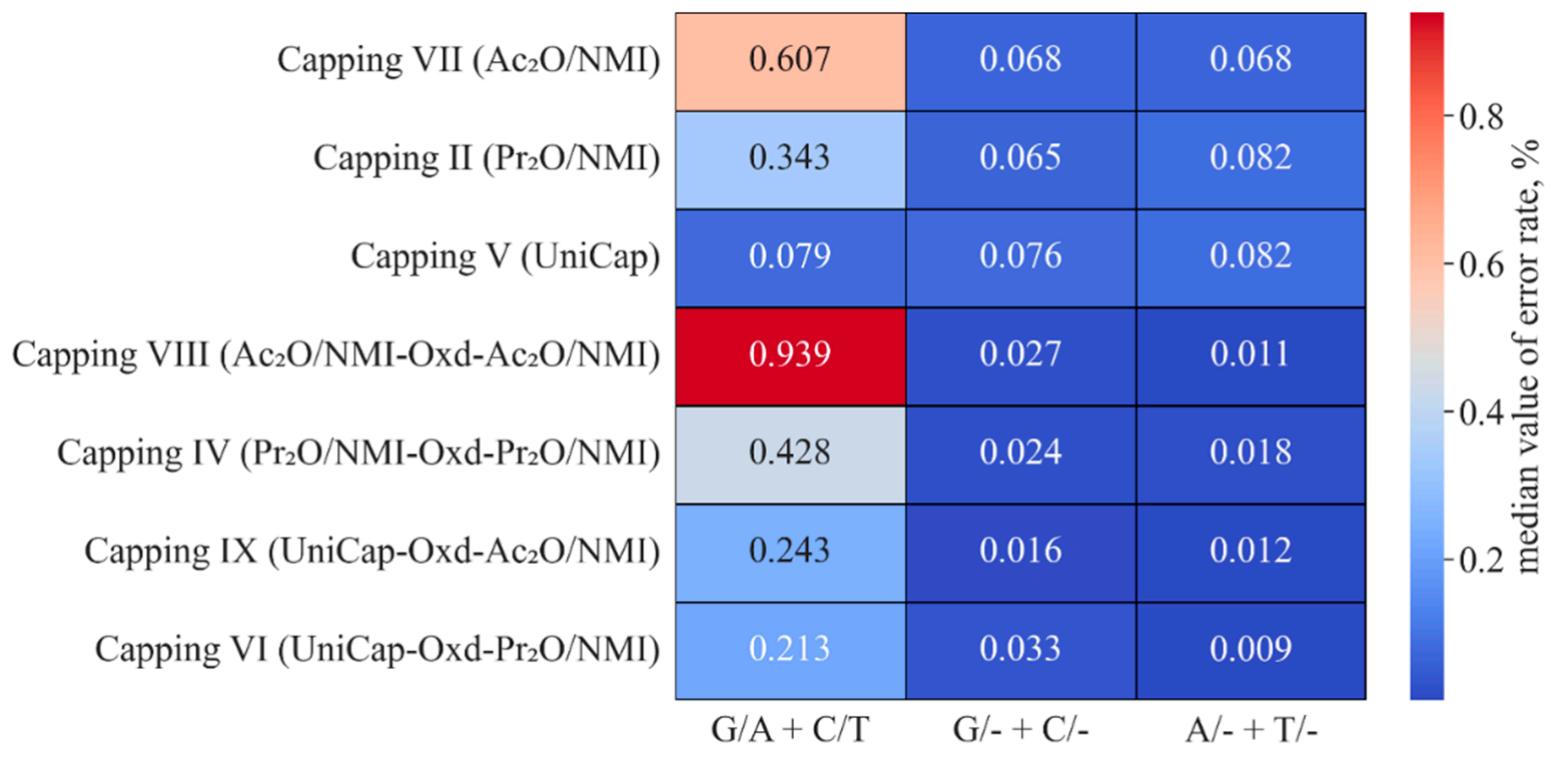
Error rates for substitution type G/A + C/T and deletions for 50 bp duplexes. The duplexes consisted of oligonucleotides, which were synthesized using the same capping conditions: Capping II or Capping IV–IX.

Consequently, 50-mer oligonucleotides were synthesized under seven distinct capping conditions. An analysis of NGS data from the corresponding duplexes revealed a pattern consistent with our previous experiment: variations in capping conditions exclusively influenced the frequencies of G/A + C/T substitutions and deletions (Supplementary File S1.Figures S1 and S2, Supplementary File S3). Moreover, the proportion of G/A + C/T substitutions varied significantly depending on the specific composition of the capping mixture (Figure 6 and Supplementary File S1.Figure S2).

Among the single-step capping protocol (conditions II, V, and VII in Figure 6), the highest frequency of G/A + C/T substitutions was observed with the Ac_2_O/NMI-based mixture (condition VII, Figure 6), yielding a median value of 0.607%. Replacing acetic anhydride with propionic anhydride (condition II, Figure 6) reduced the frequency of these substitutions to 0.343% (Supplementary File S1.Figure S2). Meanwhile, the level of deletion errors remained comparable between the acetic and propionic anhydrides treatments (Figure 6).

Double capping—performing both before and after the oxidation step—using either Ac_2_O/NMI or Pr_2_O/NMI mixtures significantly reduced the frequency of deletion errors compared to single-step capping protocols (compare conditions VII/II with VIII/IV in Figure 6). No difference in deletion rates was observed between conditions VIII and IV (Table 3, Figure 6). However, the introduction of an additional capping step—and consequently increased exposure to carboxylic acid anhydrides—led to an elevated median frequency of G/A + C/T substitutions. Nevertheless, these increases were not statistically significant when comparing single versus double capping for either Ac_2_O/NMI (VII vs. VIII) or Pr_2_O/NMI (IV vs. II). Notable, the second capping step further amplified the performance gap between the two anhydrides (Supplementary File S1.Figure S2).

These findings indicate that the use of carboxylic acid anhydrides of less acidic carboxylic acids is preferable for capping. As demonstrated, propionic anhydride maintains the effective elimination of N − 1 sequences during synthesis, while the associated increase in G/A + C/T substitution frequency is less pronounced than that observed with acetic anhydride.

Switching to an alternative capping method based on phosphoramidite chemistry (Capping V, Table 3) did not result in the pronounced increase in G/A + C/T substitutions observed with carboxylic acid anhydrides (Figure 6, Supplementary File S1.Figure S2 and Supplementary File S3). The median frequency of these substitutions using UniCap Phosphoramidite (Capping V) was 0.079%, which is 4-fold lower than with Pr_2_O/NMI (Capping II) and 7.5-fold lower than with Ac_2_O/NMI (Capping VII). Notably, the level of deletion errors with UniCap remained comparable to that of anhydride-based protocols (Figure 6).

The introduction of a second capping step—using either acetic (Capping IX, Table 3) or propionic (Capping VI, Table 3) anhydride—significantly increased the median frequency of G/A + C/T substitutions from 0.079% (Capping V) to 0.243% (Capping IX) and 0.213% (Capping VI) (Supplementary File S1.Figure S2). Nevertheless, this hybrid capping approach significantly reduced the frequency of deletion errors (Figure 6).

It has been previously hypothesized that guanosine modification arises from a side reaction between the activated phosphoramidite and the *O*^6^-atom of guanine [24], where subsequent capping and ammonolysis lead to the formation of the DAP nucleoside. However, our data indicate that carboxylic acid anhydride-based capping mixtures are independently capable of reacting with the guanine base. This is evidenced by the increase in G/A + C/T substitution frequencies in two key scenarios: (1) when a hybrid approach was employed—initial capping with UniCap followed by treatment with acetic or propionic anhydride after oxidation—and (2) when the substitution profile shifted specifically in response to the change from acetic to propionic anhydride.

While the precise mechanism of guanine modification during oligonucleotide synthesis remains speculative—given the limitations of monitoring transient intermediates during automated solid-phase synthesis—our results provide strong evidence that components of carboxylic acid anhydrides-based capping mixtures react directly with *N*^2^-protected guanosine. This interaction likely involves the *O*^6^-position, leading to the observed permanent modifications in the final product.

## 3. Materials and Methods

### 3.1. Chemistry

For oligonucleotides up to 50 nucleotides in length, Universal CPG 1000Å (Biosset, Novosibirsk, Russia) was used as the solid support; for 107-mer oligonucleotides—Universal CPG 2000Å (Glen Research, Sterling, VA, USA). Deprotection of the 5′-dimethoxytrityl group was carried out using 3% (*v*/*v*) solution of dichloroacetic acid (>99%, Sigma-Aldrich, St. Louis, MA, USA) in toluene (>99%, Reakhim, Moscow, Russia). For coupling step, 0.45 M solution of 5-ethylthio-1H-tetrazole (>99%, ChemGenes, Wilmington, MA, USA) in anhydrous acetonitrile (HPLC grade, Fisher Chemical, Pittsburgh, PA, USA) and 0.1 M solutions of phosphoramidite monomers (*N*^2^-isobutyryl-5′-*O*-(4,4′-dimethoxytrityl)-2′-deoxyguanosine-3′-*O*-[*O*-(2-cyanoethyl)-*N,N*′-diisopropylphosphoramidite], *N*^6^-benzoyl-5′-*O*-(4,4′-dimethoxytrityl)-2′-deoxyadenosine-3′-*O*-[*O*-(2-cyanoethyl)-*N*,*N*′-diisopropylphosphoramidite], *N*^4^-acetyl-5′-*O*-dimethoxytrityl-(4,4′-dimethoxytrityl)-2′-deoxycytidine-3′-*O*-[*O*-(2-cyanoethyl)-*N*,*N′*-diisopropylphosphoramidite] and 5′-*O*-dimethoxytrityl-(4,4′-dimethoxytrityl)-2′-deoxythymidine-3′-*O*-[*O*-(2-cyanoethyl)-*N*,*N′*-diisopropylphosphoramidite] (all >98% purity, Sigma-Aldrich, USA)) in acetonitrile were used. Capping of unreacted 5′-hydroxyl groups was performed using a mixture of propionic anhydride (99%, Acros Organics, Wilmington, MA, USA) or acetic anhydride (≥99%, Sigma-Aldrich, USA) in the presence of 4-dimethylaminopyridine (99%, Acros Organics, USA) or *N*-methylimidazole (99%, Alfa Aesar, Haverhill, MA, USA). Also, Unicap Phosphoramidite (Glen Research, USA) was used as an alternative capping reagent. Solvents for capping were either acetonitrile or tetrahydrofuran (>99.5%, Panreac Applichem, Castellar del Vallès, Spain). Oxidation of the phosphite triester was achieved using a solution of 0.02 M iodine (Reakhim, Russia) in pyridine (99%, Panreac Applichem, Spain) and deionized water (milli-Q grade), with tetrahydrofuran serving as the solvent carrier. Following synthesis, oligonucleotides were cleaved from the solid support and deprotected using a 40% aqueous methylamine solution (>99%, Acros Organics, USA) and 30% aqueous ammonia (Reakhim, Russia). Other chemicals were supplied by Merck (Darmstadt, Germany), Acros Organics (USA), and Reakhim (Russia).

### 3.2. Oligonucleotide Synthesis

Oligodeoxyribonucleotides were synthesized on an automatic ASM-2000 DNA/RNA synthesizer (Biosset, Russia) at a 0.2-µmol scale using 2′-deoxyphosphoramidites and solid-phase phosphoramidite synthesis protocols [28], optimized for the synthesizer (Table 1, Table 2 and Table 3). The oligonucleotides were cleaved from the solid support, and the protective groups were removed from the nucleobases by treatment with AMA mixture (30% ammonia hydroxide/40% methylamine mixture, 1:1 *v*/*v*) for 80 min at 25 °C, followed by 120 min at 45 °C. The crude oligonucleotides were then precipitated with ethanol in the presence of 0.3 M NaCl. The resulting precipitate was washed with ethanol, dried, and dissolved in 100 µL of deionized water.

The sequences of the oligodeoxyribonucleotides used in this study are listed in (Table 4 and Supplementary File S1.Table S1).

### 3.3. Purification of Oligonucleotides and Duplexes

Oligonucleotides (olig_107_1 and olig_107_2) and 107 bp duplexes were purified by PAGE. For purification of 107-mer single-stranded oligonucleotides, the following denaturing conditions were used: 12% gel (acrylamide:bisacrylamide = 19:1) containing 8 M urea, 89 mM Tris-borate (pH 8.3), and 2 mM Na_2_EDTA, electrophoresis was performed at 50 V/cm. For 107 bp duplexes purification, identical gel compositions but without urea were applied and the voltage was 40 V/cm. Gel pieces containing oligonucleotide material were incubated in water for 12 h. The resulting aqueous eluates of the target oligonucleotide fractions were concentrated to dryness and precipitated with ethanol as Na^+^ salts.

### 3.4. Duplex Assembly

Duplexes were obtained by annealing of equimolar amounts of complementary strands. The buffer used for annealing contained 50 mM NaCl, 10 mM Tris-HCl and 10 mM MgCl_2_, pH 7.9 at 25 °C. The mixture of two oligonucleotides in buffer was heated to 95 °C for 5 min and then gradually cooled to 20 °C at a rate of 2.0 °C/min using a thermal cycler.

### 3.5. Assembly of T5 Exonuclease Gene

The assembly of T5 exonuclease gene was performed by polymerase chain assembly (PCA) using a set of oligonucleotides (Supplementary File S1.Table S1). All thermocycling protocols had fixed conditions of a 2 min hot start at 98 °C, denaturation of 98 °C for 20 s, extension of 72 °C for 20 s, and a final extension at 72 °C for 3 min. The number of cycles and annealing conditions were varied.

For PCA, a mix containing a solution of oligonucleotides (Supplementary File S1.Table S1) 4 µL (0.1 µM of each oligonucleotide strand), 4 µL of 5× GC buffer, 2 µL of dNTP, 1 µL of Mg^2+^ (50 mM), 8.75 µL of nuclease-free water and 0.25 µL of Phusion High-Fidelity DNA Polymerase (2 U/µL, New England BioLabs, Ipswich, MA, USA) was prepared. Annealing conditions for PCA were 60 °C for 30 s and 25 cycles.

Follow-up PCRs were prepared with 1 µL of PCA mix, 4 µL of 5× GC buffer, 1 µL of each primer (10 µM) 0.4 µL of dNTP, 12.4 µL of nuclease-free water and 0.2 µL of Phusion High-Fidelity DNA Polymerase (2 U/µL, New England BioLabs, USA). Annealing conditions for PCR were 56 °C for 30 s and 30 cycles.

### 3.6. Cloning and Sequencing of Synthetic T5 Exonuclease Gene

Synthetic gene constructs were cloned into the *pUC19* vector using a traditional cloning workflow. Amplified T5 exonuclease gene and vector were digested with 20U of each restriction enzyme (Sybenzyme, Novosibirsk, Russia). The linearized vector was treated with 10U thermolabile alkaline phosphatase (Sybenzyme, Russia). DNA fragments with sticky ends were purified with “DNA isolation kit for Isolation of DNA from Reaction Mixtures” (Biolabmix, Novosibirsk, Russia). For ligation, the vector and gene fragment were mixed in a 1:3 molar ratio. The sticky ends were ligated using 100 U of T4 ligase (Evrogene, Moscow, Russia).

Five microliters of the cloning product were transformed into *E. coli* NovaBlue (Merk, Germany) electrocompetent cells. A BioRad MicroPulser Electroporator (Hercules, CA, USA) was used for electroporation. The procedure was performed in 1 mm cuvettes and with a standard Ec1 program (1.8 kV, 2.5 ms).

Cells were grown on agar plates with 100 µg/mL ampicillin for 16 h and then white colonies were collected for screening. Colony screening was carried out with PCR using M13 primers. Colonies containing T5 exonuclease gene insert were analyzed by Sanger sequencing, which was carried out at the Genomics Core Facility (ICBFM SB RAS, Novosibirsk, Russia).

### 3.7. NGS 

To prepare NGS libraries, oligonucleotide duplexes were phosphorylated with polynucleotide kinase, dA-tailed, and ligated to NGS adapters followed by the inclusion of index and sequencing adaptors with PCR, as was described earlier [29]. NGS libraries were pooled in equimolar concentrations and sequenced with MiniSeq Mid-Output kit (Illumina, San Diego, CA, USA) 300 cycles and MiniSeq Illumina device (Illumina, San Diego, CA, USA).

### 3.8. Processing of NGS Results

NGS reads were mapped onto the reference nucleotide sequences with bwa version 0.7.17-r1188 “mem” command [30] followed by SAM to BAM file conversion with picard (version 2.0.1) “AddOReplaceReadGroups” command. Positions and types of the mismatches, insertions, and deletions were registered with in-house Python (3.13.5) scripts, considering only events observed in both NGS reads (R1 and R2) simultaneously. Since NGS and DNA-polymerase accuracies (both values are about 99.99%) are much higher than the oligonucleotide synthesis accuracy (about 95–99%), most of the differences between reference sequence and NGS read were likely to be due to errors caused by chemical oligonucleotide synthesis.

### 3.9. Statistics

Data are expressed as the median or, if not specified, as the mean ± SD for at least three independent experiments.

The relative error frequency per 1 kb (*f*) was calculated using the number of sequenced clones (*n*), the number of errors in clone *i* (*x_i_*), and the length of sequence data from clone *i* (*l_i_*):

$$f=\;\frac{\sum_{i=1}^{n}x_{i}\frac{1000}{i}}{n}$$

To estimate the number of clones required for screening to obtain at least one error-free clone with 90% probability, the experimental data were fitted to a Poisson distribution.

Every pair of errors, obtained with NGS, at each position was counted and divided by two, because we analyzed two chains simultaneously, and by the total number of reads, and was then multiplied by 100 to calculate the error rates, %. Subsequent statistical analysis was performed using the Mann–Whitney U test.

## 4. Conclusions

Taken together, the data obtained in this study, alongside previous reports [22,23,24], demonstrate unequivocally that side reactions involving carboxylic acid anhydrides during the capping step lead to the modification of guanine residues during solid-phase phosphoramidite oligonucleotide synthesis.

It should be noted that, for many applications of synthetic oligonucleotides, the observed level of G/A + C/T errors may be negligible or non-critical. However, even these low error rates can negatively impact the quality of assembled gene constructs. Furthermore, subsequent detection and correction of errors in final products significantly increase both the duration and overall cost of gene assembly processes.

Unfortunately, precisely elucidating the side reactions mechanism remains challenging due to the multi-stage nature of the chemical synthesis process and the low frequency of substitutions. Nevertheless, strategies to minimize such errors are evident. In particular, the use of deoxyguanosine phosphoramidite bearing an additional protecting group at the *O*^6^-position should substantially reduce G/A + C/T substitutions. This hypothesis is supported by the work of Masaki et al. [22], where the implementation of 7-deazaguanosine and 8-aza-7-deazaguanosine phosphoramidites—which lack the reactive *O*^6^-carbonyl environment—reduced G/A + C/T error rates by 10- and 50-fold, respectively.

In the absence of commercially available nucleoside phosphoramidites with additional heterocyclic base protection, a practical solution to mitigate G/A + C/T substitution rates is to replace the standard acetic anhydride-based capping mixture with anhydrides of less acidic carboxylic acids. Further reduction in error can be achieved by transitioning to capping reagents based on phosphoramidite chemistry, such as UniCap.

## Figures and Tables

**Table 1:** The protocol of solid-phase oligonucleotide synthesis used in this work.

Stage of Synthetic Cycle	Reagent	Composition *	Duration
Deblocking	Dbl	3% dichloroacetic acid in toluene	50 s
Coupling	Act	0.45 M 5-ethylthio-1H-tetrazole in AcN	215 s
	Mon	0.1 M phosphoramidite nucleoside in AcN	
Capping	CapA	15% propionic anhydride in THF	210 s
	CapB	16% NMI in THF	
Oxidation	Oxd	0.02 M I_2_ in THF/Py/H_2_O 88/10/2 (*v*/*v*/*v*)	88 s
Out of synthetic cycle			
Cleavage from CPG and elimination of base labile protective groups	AMA	40% aq. methylamine/30% aq. ammonia	200 min

* AcN—acetonitrile; NMI—N-methylimidazole; Py—pyridine; THF—Tetrahydrofuran.

**Table 2:** Capping conditions for the synthesis of 107-nt nucleotides.

Capping Type	Reagent	Composition *	Duration per Cycle
Capping I	CapA	THF	210 s
	CapB	THF	
Capping II	CapA	15% propionic anhydride in THF (*v*/*v*)	210 s
	CapB	16% *N*-methylimidazole in THF (*v*/*v*)	
Capping III	CapA	15% propionic anhydride in THF (*v*/*v*)	210 s
	CapB	6.5% DMAP in THF (*m*/*v*)	

* THF—Tetrahydrofuran.

**Table 3:** Capping conditions for synthesis of 50 nt oligonucleotides.

Capping Type	Conditions of Capping Steps *
Capping II	Cycle contained one capping before oxidation step. Capping mixture: 15% Pr_2_O/16% NMI in THF (1:1 *v*/*v*) were used; contact time was 210 s.
Capping IV	Cycle contained two capping steps: before and after oxidation step. Capping mixture: 15% Pr_2_O/16% NMI in THF (1:1 *v*/*v*) were used. The first cap lasted 210 s, second—105 s.
Capping V	Cycle contained one capping before oxidation step. Capping mixture: 0.1 M UniCap/0.45 M ETT in AcN (1:1 *v*/*v*) were used; contact time was 225 s.
Capping VI	Cycle contained two capping steps: before and after oxidation step. For the first capping 0.1 M UniCap/0.45 M ETT in AcN (1:1 *v*/*v*) were used. Capping mixture: 15% Pr_2_O/16% NMI in THF (1:1 *v*/*v*) were used for second cap. The first cap lasted 225 s, second—105 s.
Capping VII	Cycle contained one capping before oxidation step. Capping mixture: 10% Ac_2_O/16% NMI in THF (1:1 *v*/*v*) were used; contact time was 210 s.
Capping VIII	Cycle contained two capping steps: before and after oxidation step. Capping mixture: 10% Ac_2_O/16% NMI in THF (1:1 *v*/*v*) were used. The first cap lasted 210 s, second—105 s.
Capping IX	Cycle contained two capping steps: before and after oxidation step. For the first capping 0.1 M UniCap/0.45 M ETT in AcN (1:1 *v*/*v*) were used. Capping mixture: 10% Ac_2_O/16% NMI in THF (1:1 *v*/*v*) were used for second cap. The first cap lasted 225 s, second—105 s.

* AcN—acetonitrile; ETT—5-ethylthio-1H-tetrazole; NMI—N-methylimidazole; THF—tetrahydrofuran.

**Table 4:** Sequences of oligodeoxyribonucleotides synthesized with ASM-2000.

Oligonucleotide	Sequence 5′-3′
olig_107_1	GGCAGCGTGCAGCTCGCCGACCACTACCAGCAGAACACCCC CATCGGCGACGGCCCCGTGCTGCTGCCCGACAACCACTACCT GAGCACCCAGTCCGCCCTGAGCAA
olig_107_2	TTGCTCAGGGCGGACTGGGTGCTCAGGTAGTGGTTGTCGGG CAGCAGCACGGGGCCGTCGCCGATGGGGGTGTTCTGCTGGT AGTGGTCGGCGAGCTGCACGCTGCC
olig_50_1	CTAATCAGTTCAGCAGACTCCAGTAGTCTAAGTTCGGAAAGC AATGGCGT
olig_50_2	ACGCCATTGCTTTCCGAACTTAGACTACTGGAGTCTGCTGAA CTGATTAG

## Data Availability

The original contributions presented in this study are included in the article/Supplementary Material. Further inquiries can be directed to the corresponding author.

## Supplementary Materials

The following supporting information can be downloaded at: https://www.mdpi.com/article/10.3390/molecules31010094/s1, Supplementary File S1: Fasta S1—sequence of T5_exonuclease gene, Table S1—oligonucleotides for assembly of T5_exonuclease gene by PCA, Figure S1—distribution of error probabilities depending on capping conditions for 50 bp duplexes, Figure S2: Statistical analysis (*U*-test) of the G/A + C/T error rate depending on the type of capping conditions, *-*p*-value < 0.05; **-*p*-value < 0.005; ***-*p*-value < 0.0005. Supplementary File S2: processed Sanger sequencing data, Supplementary File S3: processed NGS data.
