# Supplementary material for: The Influence of the Capping Step During Solid-Phase Phosphoramidite Synthesis of Oligonucleotides on Synthetic Errors in Oligonucleotides
Source: Molecules. 2025 Dec 25;31(1):94. doi: 10.3390/molecules31010094 (PMC12786494; doi:10.3390/molecules31010094)

S1.Fasta S1: Sequence of T5_exonuclease gene

>T5_exonuclease

AGGACTTAATTAAATAATGAGTAAATCCTGGGGAAAATTTATTGAAGAAGAGGAAG
CTGAAATGGCTTCCCGTCGTAATCTAATGATTGTCGATGGAACTAACTTAGGCTTTC
GCTTCAAACATAACAATAGTAAAAAACCATTTGCCTCAAGTTATGTTTCAACTATTC
AATCTCTGGCAAAATCCTACTCTGCCAGAACTACGATTGTTCTAGGTGATAAGGGAA
AATCCGTATTTCGTCTAGAACATCTACCAGAGTATAAAGGTAATCGTGATGAAAAGT
ACGCACAACGTACGGAAGAGGAGAAAGCGCTAGATGAGCAGTTCTTTGAGTATTTG
AAGGATGCTTTCGAGTTGTGTAAAACTACATTCCCAACTTTTACCATTCGTGGTGTA
GAAGCAGACGATATGGCAGCTTATATTGTTAAGCTCATCGGGCATCTTTATGATCAC
GTTTGGCTAATATCTACAGATGGTGACTGGGATACTTTATTAACGGATAAAGTTTCT
CGTTTTTCTTTCACAACACGTCGTGAGTATCATCTTCGTGATATGTATGAACATCATA
ATGTTGATGATGTTGAGCAGTTTATCTCCCTGAAAGCAATTATGGGAGATCTAGGAG
ATAATATTCGTGGTGTTGAAGGAATAGGAGCAAAACGCGGATATAATATTATTCGT
GAGTTTGGTAACGTACTGGATATTATTGATCAGCTTCCACTGCCTGGAAAGCAGAAA
TATATACAGAACCTGAATGCATCGGAAGAACTGCTTTTCCGAAACTTGATTCTGGTT
GATTTACCTACCTACTGTGTGGATGCTATTGCTGCTGTAGGTCAAGATGTGTTAGAT
AAGTTTACAAAAGATATTTTGGAGATTGCAGAACAATGA

S1.Table S1: oligonucleotides for assembly of T5_exonuclease gene by PCA

T5_exo_1	AGGACTTAATTAAATAATGAGTAAATCCTGGGGAAAA
T5_exo_2	ACGACGGGAAGCCATTTCAGCTTCCTCTTCTTCAATAAATTTTCCCCAGGATTTACTCAT
T5_exo_3	ATGGCTTCCCGTCGTAATCTAATGATTGTCGATGGAACTAACTTAGGCTTTCGCT
T5_exo_4	ACTTGAGGCAAATGGTTTTTTACTATTGTTATGTTTGAAGCGAAAGCCTAAGTTAGT
T5_exo_5	AAAACCATTTGCCTCAAGTTATGTTTCAACTATTCAATCTCTGGCAAAATCCTACTCT
T5_exo_6	CGGATTTTCCCTTATCACCTAGAACAATCGTAGTTCTGGCAGAGTAGGATTTTGCCAGAG
T5_exo_7	AGGTGATAAGGGAAAATCCGTATTTCGTCTAGAACATCTACCAGAG
T5_exo_8	CGTACGTTGTGCGTACTTTTCATCACGATTACCTTTATACTCTGGTAGATGTTCTAGACG
T5_exo_9	AGTACGCACAACGTACGGAAGAGGAGAAAGCGCTAGATGAGCAG

T5_exo_10	AGTTTTACACAACTCGAAAGCATCCTTCAAATACTCAAAGAACTGCTCATCTAGCGCT
T5_exo_11	GCTTTCGAGTTGTGTAAAACTACATTCCCAACTTTTACCATTCGTGGTGTAGAAGCAG
T5_exo_12	AGATGCCCGATGAGCTTAACAATATAAGCTGCCATATCGTCTGCTTCTACACCACGAA
T5_exo_13	AGCTCATCGGGCATCTTTATGATCACGTTTGGCTAATATCTACAGATGGTGACTGGGA
T5_exo_14	AAAACGAGAAACTTTATCCGTTAATAAAGTATCCCAGTCACCATCTGT
T5_exo_15	ACGGATAAAGTTTCTCGTTTTTCTTTCACAACACGTCGTGAGTATCATCTTCGTGA
T5_exo_16	AACTGCTCAACATCATCAACATTATGATGTTCATACATATCACGAAGATGATACTCACG
T5_exo_17	GTTGATGATGTTGAGCAGTTTATCTCCCTGAAAGCAATTATGGGAGA
T5_exo_18	GCTCCTATTCCTTCAACACCACGAATATTATCTCCTAGATCTCCCATAATTGCTTTCAGG
T5_exo_19	GGTGTTGAAGGAATAGGAGCAAAACGCGGATATAATATTATTCGTGAGTTTGGTAACGT
T5_exo_20	CTGCTTTCCAGGCAGTGGAAGCTGATCAATAATATCCAGTACGTTACCAAACTCACGAAT
T5_exo_21	ACTGCCTGGAAAGCAGAAATATATACAGAACCTGAATGCATCGGAAGAACTGCTTTTCC
T5_exo_22	ATCCACACAGTAGGTAGGTAAATCAACCAGAATCAAGTTTCGGAAAAGCAGTTCTTCCGA
T5_exo_23	ACCTACCTACTGTGTGGATGCTATTGCTGCTGTAGGTCAAGATGTGTTAGATAAGT
T5_exo_24	TCATTGTTCTGCAATCTCCAAAATATCTTTTGTAAACTTATCTAACACATCTTGACCT

S1.Figure S1: Distribution of error probabilities depending on capping conditions for 50 bp duplexes

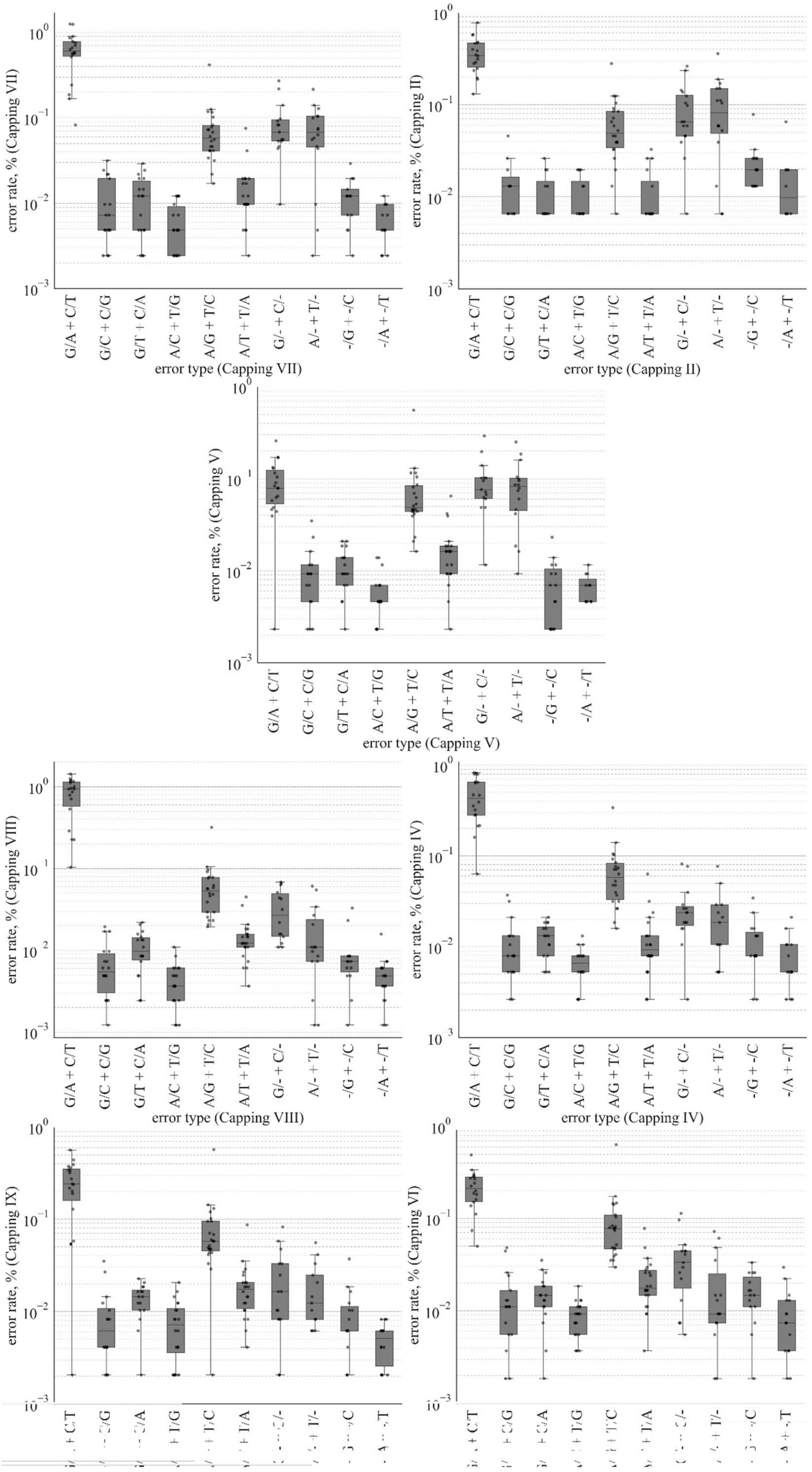

error rate, % (Capping VII)
error type (Capping VII)
error rate, % (Capping II)
error type (Capping II)
error rate, % (Capping V)
error type (Capping V)
error rate, % (Capping VIII)
error type (Capping VIII)
error rate, % (Capping IV)
error type (Capping IV)
error rate, % (Capping IX)
error rate, % (Capping VI)
G/A + C/T
G/C + C/G
G/T + C/A
A/C + T/G
A/G + T/C
A/T + T/A
G/- + C/-
A/- + T/-
-/G + -/C
-/A + -/T

S1.Figure S2: Statistical analysis (*U*-test) of the G/A + C/T error rate depending on the type of capping conditions, * - *p*-value < 0.05; ** - *p*-value < 0.005; *** - *p*-value < 0.0005

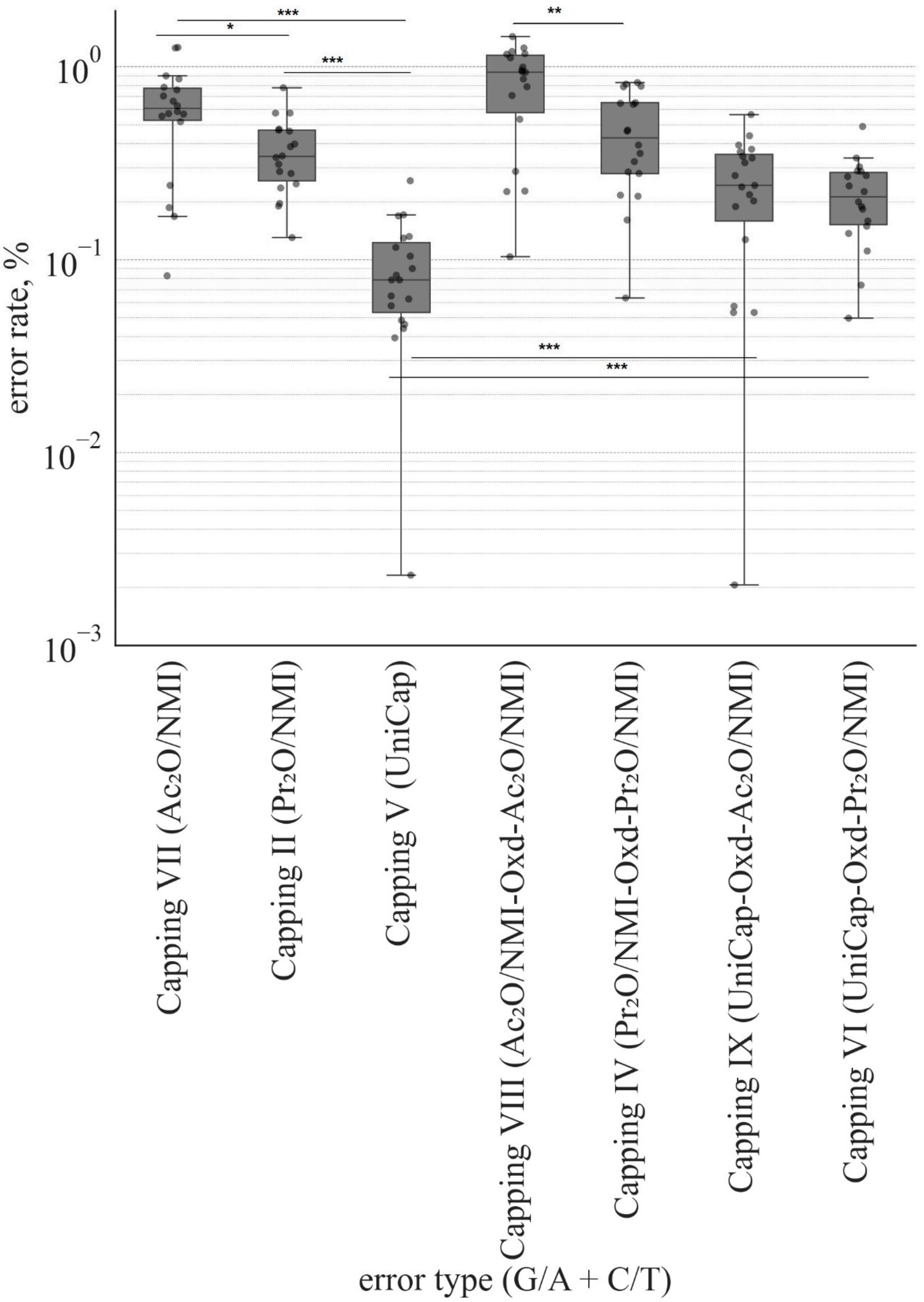

Supplement: Supplementary file 1 [file molecules-31-00094-s001.zip › Suplimentary_S1.pdf]
